# Comprehensive Gene Panel Analysis of Biliary Tract Cancer Using Next-Generation Sequencing of Endoscopic Transpapillary Brushing/Biopsy/Aspiration Specimens: A Narrative Review

**DOI:** 10.3390/diagnostics16101516

**Published:** 2026-05-16

**Authors:** Masaki Kuwatani, Naoya Sakamoto

**Affiliations:** Department of Gastroenterology and Hepatology, Hokkaido University Hospital, North 14, West 5, Kita-ku, Sapporo 060-8648, Japan; sakamoto@med.hokudai.ac.jp

**Keywords:** genomic profiling, brushing, biopsy, bile, mutation, biliary tract cancer

## Abstract

The undesired prognosis of biliary tract cancer is mainly attributed to the difficulty in detecting cancer lesions, including intraepithelial neoplasia, and other hurdles in procuring sufficient pathological samples by forceps biopsy and brushing, or even their combination. However, the transpapillary approach under endoscopic retrograde cholangiopancreatography (ERCP) is the mainstream approach for the work-up and treatment of biliary tract diseases, especially biliary tract cancers, because the ERCP-guided approach efficiently enables simultaneous biliary drainage for the treatment of cholangitis/jaundice and specimen acquisition for the diagnosis of biliary tract lesions. To improve diagnostic accuracy, several studies have been conducted on the feasibility and efficacy of genomic analysis of endoscopic specimens, namely, brushing samples, forceps biopsy samples, and aspiration samples such as bile with sensitivities ranging from 47 to 100%, with specificities ranging from 69 to 100%. Clinical use of genomic analysis remains heterogeneous due to the panel and next-generation sequencing system. For the efficient and precise treatment of patients with biliary tract cancer, future diagnosis and treatment should be based on molecular and genetic analyses. In this article, we review and summarize the comprehensive gene panel analyses of transpapillary brushing/biopsy/aspiration specimens for biliary tract cancer using next-generation sequencing, promoting effective clinical practice and providing a basis for future studies.

## 1. Introduction

The disastrous prognoses of biliary tract cancers (BTCs) comprising cholangiocarcinoma and gallbladder cancer—5-year relative survival rates of 24.5–28.9% in 2025 in Japan [1] and 10–21.2% in the U.S. [2]—are caused by some hurdles to early diagnosis and procurement of sufficient pathological samples. The main obstacles are difficult target locations for observation and narrow spaces for the procurement of pathological specimens under endoscopy. The transpapillary approach under endoscopic retrograde cholangiopancreatography (ERCP) is the mainstream approach for work-up and treatment of biliary tract diseases, especially BTCs, although it also harbors risk of post-ERCP pancreatitis ranging from 5% to 10%, which can lead to lethal complications, such as cholangitis ranging from 0.5 to 3.0%, cholecystitis ranging from 0.5 to 5.2%, and death attributable to ERCP ranging from 0 to 0.4% in terms of occurrence rates [3,4,5]. When ERCP for patients with surgically altered anatomy is difficult or impossible due to intestinal adhesions or a long afferent loop, brushing/forceps biopsy via a percutaneous approach is also available for acquisition of specimens. Furthermore, forceps biopsy under cholangioscopy could occasionally yield effective specimens [6]. However, because the ERCP-guided approach efficiently enables simultaneous biliary drainage for the treatment of cholangitis or jaundice and specimen acquisition for the diagnosis of biliary malignancy/benignancy, it has long been a first-line method in the field of BTC practice. On the other hand, the diagnostic abilities of forceps biopsy with/without brushing cytology for malignant biliary strictures during ERCP-guided procedures have been reported to have a sensitivity of 40–60% and specificity of 97–100% in biopsy alone, whereas they have been reported to have a sensitivity of 47–86% and specificity of almost 100% in biopsy with cytology [7,8,9,10,11,12,13,14]. Low sensitivity is a problem with biliary forceps biopsy with/without cytology.

Although there is still no curative therapy other than radical surgical resection and the conventional standard chemotherapies for unresectable BTC have not yielded preferable outcomes (median survival times of 11.7 and 13.5 months) [15,16], recent highly targeted treatment or immunotherapies such as FGFR inhibitors and immune checkpoint inhibitors (PD-1/PD-L1/CTLA-4 inhibitors) have achieved longer survival: recent reports regarding targeted therapies (FIGHT-202 trial with pemigatinib, FGFR inhibitor/TOPAZ-1 trial with durvalumab, PD-L1 inhibitor) demonstrated that overall survival (OS) and progression-free survival were 17.5/12.8 months and 7/7.2 months, respectively [17,18,19]. Furthermore, adjuvant chemotherapy for BTC with S-1 (ASCOT trial)/capecitabine (BILCAP trial) indicated that OS and relapse-free survival rates were 5.2 years or more/51.1 months and 5.3 years/24.4 months, respectively, which were significantly longer periods than surveillance alone after surgery [20,21]. For efficient and personalized medicine for patients with both resectable and unresectable BTCs, future diagnosis and treatment should be based on comprehensive molecular and genetic analyses of biopsy, cytology, and bile specimens via a minimally invasive method. Moreover, in the last few decades, technologies for genetic analyses, including next-generation sequencing (NGS) with high speed and low cost, third- or fourth-generation sequencing with long reads, and multiplex droplet digital PCR with high sensitivity, have emerged and rapidly spread [22,23].

In this article, we review and summarize the current status of comprehensive genetic analyses for BTC using NGS with endoscopic transpapillary brushing/biopsy/aspiration specimens for feasible precision medicine and future research on diagnosis and treatment.

For this narrative review, the complete PubMed search query was as follows: (“endoscopic retrograde cholangiopancreatography” [Title/Abstract] OR “ERCP” [Title/Abstract]) AND (“brushing” [Title/Abstract] OR “biopsy” [Title/Abstract] OR “bile” [Title/Abstract] OR “next generation sequencer” [Title/Abstract] OR “next generation sequencing” [Title/Abstract] OR “NGS” [Title/Abstract]) OR “NGS” [Title/Abstract] OR “comprehensive genome profiling” [Title/Abstract] OR “genome profiling” [Title/Abstract]) with the time frame “2010–2025”.

## 2. Brushing Specimens

In general, it is difficult to obtain sufficient specimens from a biliary stricture site for pathological diagnosis and genetic analysis via the transpapillary route because devices cannot function in such narrow spaces. Because a brushing device is relatively easy to advance through and hit the stricture site, it is frequently used, particularly in the intrahepatic bile duct. However, brushing specimens are usually tiny cell agglomerates that are difficult to evaluate histologically using standard methods. According to the meta-analysis by Navaneethan et al., the pooled sensitivity and specificity of brushings for the diagnosis of malignant biliary strictures was 45% (95% confidence interval [CI], 40–50%) and 99% (95% CI, 98–100%), respectively [11]. Another recent meta-analysis indicated that final malignancy risks in 2826 initial indeterminate samples with atypical and suspicious brush cytology were 50.4% and 80.2%, respectively [24]. Finally, there was a 60.1% risk of malignancy among all samples with indeterminate brush cytology. To overcome this intractable situation, genetic analyses of brushing samples have been conducted using NGS (Table 1 and Table S1).

### 2.1. Diagnostic Ability and Accuracy Based on Genetic Analysis

Eight previous studies investigated the diagnostic performance of NGS of brushing samples in the field of BTC (Table 1 and Table S1) [25,26,27,28,29,30,31,32]. These studies included patients with bile duct strictures due to malignancy and benignancy, with a total of 169 malignant/289 benign strictures, and assessed their genetic alteration profiles. The success rates of DNA extraction were 61.5–100%, and the adjusted amount of the extracted DNA was varied from 0.08 to 27.45 ng/μL (mean, 3.17 ng/μL; median, 1.61 ng/μL) [26], or a DNA input was pursued up to 50 ng (in general, a limit of detection down to 0.1% is achieved with a DNA input of 20 ng to NGS) [30]. When one or more mutations/alterations or copy number variants of driver, passenger, or fusion genes in NGS are assumed to be malignant (positive), the sensitivities are 55, 63, 72, 75, 89, and 100%; the specificities are 73, 82, 85, 88, 89, 98, and 100%; the positive predictive values are 40, 79, 80, 86, 88, and 100%; the negative predictive values are 63, 76, 89, 98, and 100%; and the accuracies are 78, 86, 89, 90, 91, and 98%, respectively. Compared with brushing cytology alone, NGS with brushing specimens has a superior diagnostic ability for malignancy as a whole, while the results, especially the sensitivities, vary among studies. The variety of the results would depend on the gene panels used, which, respectively, contain different genes and different gene numbers (range: 28–161); the NGS instrument used with different mutation/variant allele frequencies (range: 0.1–5%); the cohort composition, such as whether it included patients with primary sclerosing cholangitis, as seen in the studies by Scheid et al. [29], Kamp et al. [30], and Boyd et al. [31]; and condition and treatment processes of the acquired specimens.

NGS can also occasionally decrease specificity because of genetic alterations in some patients with primary sclerosing cholangitis or cholelithiasis, some of whom may be candidates for BTC. A study by Arechederra et al. also highlighted intriguing findings, that final sensitivity for malignancy in the NGS assay with bile cfDNA was 100% in patients with an initial diagnosis of benign or indeterminate strictures resulting in the initial low specificity, using a 161-gene panel. In other words, its low specificity was caused by prior mutations that indicated the presence of precancerous lesions or carcinoma in situ [33].

***Brief note: Overall, NGS of brushing specimens improves sensitivity over cytology but remains heterogeneous due to the panel, NGS system and cohort variability.***

### 2.2. Detected Mutations/Alterations of Genes

In 2015, Nakamura et al. comprehensively and precisely demonstrated, with 260 BTC surgical specimens, the various genetic alterations/mutations in BTC according to the anatomical primary site of BTC, namely, intrahepatic cholangiocarcinoma (ICC), extrahepatic cholangiocarcinoma (ECC), gallbladder cancer (GC), and ampullary cancer (AC) [34]. For instance, ICC and ECC share *KRAS*, *SMAD4*, *ARID1A*, and *GNAS* mutations, whereas BTC harbors *TP53*, *BRCA1/2*, and *PIK3CA* mutations. Their report significantly promoted the NGS of transpapillary specimens.

**Figure 1:**
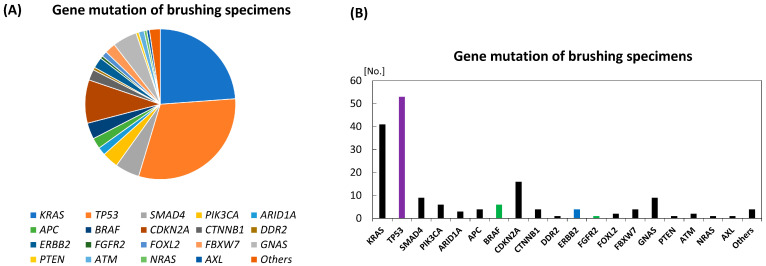
Genetic mutation/alteration spectrum revealed by brushing specimens in the previous eight studies. (**A**) Proportion of each detected genetic mutation. (**B**) Number of each detected genetic mutation. Colored bars mean OncoKB therapeutic levels: green, level 1 (FDA-recognized biomarker); blue, level 2 (standard care biomarker); and purple, level 3A (compelling clinical evidence).

Regarding brushing specimens from 134 patients with BTC, including seven with GC and seven with AC who were positive for mutation (Table 1 and Table S1) [25,26,28,29,30,31,32], about 170 gene alterations/mutations in about 20 genes were detected and the average mutation/alteration number per tumor was 1.7 (229/134), which is consistent with an estimated number of 2.1 mutations per tumor sample calculated based on the Cosmic database [35]. Of the 120 patients with ICC or ECC (breakdown unavailable), mutations in *TP53* and *KRAS* were more frequently detected, both of which accounted for just over 50% of the total, as well as the previous report by Nakamura et al. [34] (Figure 1). *CDKN2A*, *SMAD4* and *GNAS* were subsequently detected; as previous reports indicated, other various genes in small numbers were also detected in approximately 25% of the positive genes using gene panels that comprise 14–723 genes (Table 1 and Table S1) [34,36]. Therefore, it is difficult to detect gene alterations/mutations in BTC using gene panels that cover only a small number of genes. Meanwhile, the top seven key genes comprising *TP53*, *KRAS*, *CDKN2A*, *SMAD4*, *GNAS*, *PIK3CA*, and *BRAF* cover approximately 80% of mutated genes; in the top 10 key genes with the additional *APC*, *CTNNB1*, and *ERBB2*, the covering rate rises up to 88%, which is available and acceptable in clinical practice.

When actionable gene alterations, namely, therapeutically relevant alterations, are defined using a combination of OncoKB classification, ESMO Scale for Clinical Actionability of Molecular Targets (ESCAT), NCCN Clinical Practice Guidelines in Oncology, and FDA-approved regimens [37,38,39,40], 64 actionable alterations (*TP53*, 53 alterations; *BRAF*, 6; *ERBB2*, 4; *FGFR2*, 1), classified into level 1 (FDA-recognized biomarker), level 2 (standard care biomarker) or level 3A (compelling clinical evidence), at 37.2% (78% if alterations with biological evidences such as *KRAS* and *PIK3CA* mutations are included) of the 172 alterations were detected in the previous eight reports. If only gene alterations are detected in the brushing specimens, physicians will need to consider precision medicine based on actionable alterations in the near future.

***Potential key genes for diagnosis based on frequencies: TP53, KRAS, CDKN2A, SMAD4, GNAS, PIK3CA, and BRAF.***

***Key genes for treatment: BRAF, FGFR2, ERBB2, and TP53.***

## 3. Forceps Biopsy Specimens

Biopsy forceps cannot be opened in the stenotic bile duct because of its narrow space. Therefore, there have been several reports of combining forceps biopsy with brushing cytology and/or aspiration bile cytology for an improved pathological diagnosis, with a higher sensitivity of around 85% and specificity of around 100%, although the sensitivity is not entirely satisfactory [14].

The results of the study by Bankov et al. revealed genetic heterogeneity in biliary neoplasia in their gene set: namely, 28% of mutations were found in biopsy samples alone, but not in the corresponding surgical specimens [41]. Moreover, a slightly higher mutation rate was found in biopsy specimens than in surgical specimens, whereas the allele frequency was lower. Their data show that biliary dysplasia contains remarkable subclones and indicate that CCA could be derived from a minor subclone. Similar subclonal heterogeneity has been demonstrated in other cancers, such as esophageal adenocarcinoma, and oral precancerous lesions [42,43].

### 3.1. Diagnostic Ability and Accuracy Based on Genetic Analysis

Only four studies have investigated the diagnostic performance of NGS for forceps biopsy samples in the field of BTC (Table 2 and Table S2) [26,41,44,45]. These studies included patients with bile duct strictures due to malignancy and benignancy (147 malignant/92 benign strictures), and assessed the genetic alteration profiles of malignant and benign strictures. The mean size of forceps biopsy samples reported by Bankov et al. was 2.2 mm with a range of 0.5–8 mm (not reported in the remaining two studies) [41]. For reference, endoscopic ultrasonography-guided tissue acquisition samples with 22-gauge Franseen needles for a general comprehensive genetic profiling test (the FoundationOne^®^ CDx, F1CDx; Foundation Medicine, Inc., Cambridge, MA, USA) require 4 mm or longer in length according to a previous report by Ishiwatari et al. [46]. They also demonstrated that the acquisition rate of ideal samples for F1CDx was 96% with 100% of F1CDx success in passed samples.

The success rates of DNA extraction for research in the above-mentioned four studies ranged from 92.3% to 100%; the amounts of the extracted DNA were all >10 ng required for their gene panel tests, and the adjusted amount of the extracted DNA varied from 0.53 to 35.71 ng/μL (mean, 6.42 ng/μL; median, 4.22 ng/μL) [26]. The current gene panels with hundreds of genes (TargetGxOne™ Amplicon Sequencing by Azenta Life Sciences, Chelmsford, MA, USA; FoundationOne^®^ CDx by Foundation Medicine, Inc., Cambridge, MA, USA) require at least >100 ng and 50 ng of DNA input volume for analysis, respectively. When at least one mutation/alteration or copy number variant in NGS is assumed to be malignant, the sensitivities are 71, 83, and 88%; the specificities are 100%, respectively [41].

***Brief note: Overall, NGS of forceps biopsy specimens significantly improves sensitivity over cytology but remains uncertain due to small study number and cohorts.***

### 3.2. Detected Mutations/Alterations of Genes

**Figure 2:**
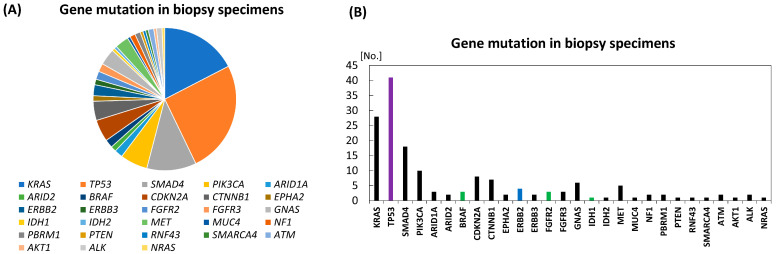
Genetic mutation/alteration spectrum revealed by forceps biopsy specimens in three previous studies. (**A**) Proportion of each detected genetic mutation. (**B**) Number of each detected genetic mutation. Colored bars mean OncoKB therapeutic levels: green, level 1; blue, level 2; and purple, level 3A.

Regarding biopsy specimens from 83 patients with BTC, including 16 with ICC, 52 with ECC, 10 with AC, three with GC, and two with high-grade dysplasia, patients with mutation positivity in the three studies (Table 2 and Table S2) [26,41,45], a total of approximately 183 gene alterations/mutations in 28 genes were detected, and the average mutation/alteration number per tumor was 2.2 (183/83) (Figure 2). Of these 83 patients, mutations in *TP53* and *KRAS* accounted for 45% of the total. *SMAD4*, *PIK3CA*, and *CDKN2A* were subsequently detected, whereas as previous reports indicated, other various genes with small numbers were also detected in approximately 30% of the positive genes using gene panels comprising 20 or 41 genes (Table 2 and Table S2). Meanwhile, the top seven key genes comprising *TP53*, *KRAS*, *SMAD4*, *PIK3CA*, *CDKN2A*, *CTNNB1* and *GNAS*, covered approximately 73% of mutated genes; in the top 10 key genes with additional *MET*, *ERBB2*, and *BRAF* or *FGFR2*, the covering rate increases to 81%, which is available and acceptable in clinical practice.

Regarding actionable gene alterations, 52 actionable alterations (*TP53*, 41 alterations; *BRAF*, 3; *ERBB2*, 4; *FGFR2*, 3) at 32.3% (68% if alterations with biological evidence such as *KRAS* and *PIK3CA* mutations are included) of the 183 alterations were detected in the three previous reports.

***Potential key genes for diagnosis based on frequencies: TP53, KRAS, SMAD4, PIK3CA, CDKN2A, CTNNB1 and GNAS.***

***Key genes for treatment: BRAF, FGFR2, IDH1, ERBB2, and TP53.***

## 4. Bile (Aspiration) Specimens

Unexpectedly, there have been eight previous reports regarding comprehensive/cancer hotspot gene panel tests with NGS in bile (aspiration) specimens, more than in forceps biopsy specimens, partially due to the greater sampling error of forceps biopsy (Table 3 and Table S3) [33,47,48,49,50,51,52,53]. The concept of NGS of bile specimens is to analyze DNA/RNA extracted from exfoliated/detached tumor cells from BTC floating in the bile or DNA directly released from BTC cells exposed to the biliary tract lumen, which can be considered as cell-free DNA. Major challenges when analyzing cfDNA of blood plasma are that the amount is low, generally less than 10 ng/mL in healthy individuals [54], while the amount of bile cfDNA is much more, ranging from 100 to 70,800 ng/mL (Table 3 and Table S3). Meanwhile, it is necessary to accurately identify the biliary lesion/stricture site before NGS of bile and to be cautious about interpreting the NGS results, considering precancerous lesions such as biliary intraepithelial lesions without abnormal imaging findings or future cancerous lesions with both normal cell forms and initial gene alterations, such as primary sclerosing cholangitis. Meanwhile, NGS of bile has an advantage in that it provides genetic information on distant sites that a brushing or biopsy forceps device cannot reach, such as the peripheral bile duct and gallbladder. Furthermore, bile can harbor more cell-free tumor-derived DNA fragments than plasma, because BTC cells directly contact bile in the biliary tract. Therefore, NGS of bile specimens is promising for both early detection of BTC and potentially high-risk lesions.

### 4.1. Diagnostic Ability and Accuracy Based on Genetic Analysis

There have been eight previous studies that investigated the diagnostic performance of NGS of bile samples in the field of BTC (Table 3 and Table S3) [33,47,48,49,50,51,52,53]. These studies included patients with bile duct strictures due to malignancy and benignancy (190 malignant and 161 benign stricture/lesions) and assessed the genetic alteration profiles of the malignant and benign strictures/lesions. The success rates of DNA extraction were all 100%, and the adjusted amount of the extracted DNA ranged from 100 to 70,800 ng/mL before processing and 2.4 to 6165 ng/μL after processing. When one or more mutations/alterations or copy number variants in NGS are assumed to be malignant (positive), the sensitivities are 47, 56, 58, 60, 70, 75, and 100%; the specificities are 66, 69, 75, 79, 96 and 100%; the positive predictive values are 13, 77, 88, 91, and 100%; the negative predictive values are 33, 50, 66, 89, 98, and –100%; and the accuracies are 60, 67, 88, 92, and 100%, respectively. Notably, in the cohort comprising patients with PSC, the specificity of 66.1% and positive predictive value of 13% for malignancy were lower than in the cohorts of other previous reports, which could reflect the existence of precancerous lesions or nondetectable carcinoma in situ in imaging modalities [33,53]. Therefore, the interpretation of genetic variants in the PSC cohort should be conducted with particular caution and from a long-term perspective, considering future cancer development.

***Brief note: Overall, NGS of bile specimens improves sensitivity over cytology, but not specificity, and remains heterogeneous due to panel, NGS system and cohort variability.***

### 4.2. Detected Mutations/Alterations of Genes

**Figure 3:**
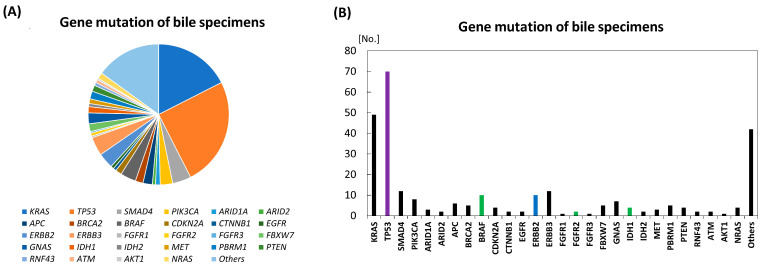
Genetic mutation/alteration spectrum revealed by bile specimens in the seven previous studies. (**A**) Proportion of each detected genetic mutation. (**B**) Number of each detected genetic mutation. Colored bars mean OncoKB therapeutic levels: green, level 1; blue, level 2; and purple, level 3A.

Regarding bile specimens from 184 patients with BTC, including 39 with GC and three with AC who were positive for mutation (Table 3 and Table S3), approximately 280 gene alterations/mutations in about 45 genes were detected and the average mutation/alteration number per tumor was 2.4 (280/115) (Figure 3) [33,47,48,49,50,51,52,53]. Of the 115 patients with BTC, mutations in *TP53* and *KRAS* accounted for just more than 40% of the total. *SMAD4, BRAF*, *ERBB2*, and *ERBB3* were subsequently detected, whereas as previous reports indicated, other various genes with small numbers were detected in approximately 42% of the positive genes using gene panels comprising 7–60 genes (Table 3 and Table S3). Therefore, it is difficult to detect gene alterations/mutations in BTC using gene panels that cover only a small number of genes. Meanwhile, the top 10 key genes comprising *TP53*, *KRAS*, *SMAD4*, *BRAF*, *ERBB2*, *ERBB3*, *PIK3CA*, *GNAS*, *APC* and *FBXW7* can cover approximately 67% of mutated genes; in the top 15 key genes with additional *BRCA2*, *NRAS*, *PBRM1*, *CDKN2A* and *CTNNB1*, the covering rate increases to 74%, which is available, but not satisfactory in clinical practice.

Regarding actionable gene alterations, 96 actionable alterations (TP53, 70 alterations; *BRAF*, 10; *ERBB2*, 10; *FGFR2*, 2; *IDH1*, 4) at 32% (53% if alterations with biological evidences such as *KRAS* and *PIK3CA* mutations are included) of the 280 alterations were detected in the seven previous reports.

***Potential key genes for diagnosis based on frequencies: TP53, KRAS, SMAD4, BRAF, ERBB2, ERBB3, PIK3CA, GNAS, APC and FBXW7.***

***Key genes for treatment: BRAF, FGFR2, IDH1, ERBB2, and TP53.***

## 5. Conclusions and Future Perspectives

Many endoscopists and physicians have been exploring methods for acquiring sufficient material for the conventional pathological diagnosis of BTC for decades; however, this exploration is currently shifting to molecular/biological diagnosis using limited and tiny specimens obtained via minimally invasive methods, leading to personalized precision medicine. As described above, although its diagnostic accuracy is still developing and its clinical use for diagnosis requires more investigation and validation with large cohorts, considering cost-effectiveness, several characteristic genomic features such as more frequent alterations of *TP53* and *KRAS* and diversity of less frequent alterations have also been revealed by NGS of brushing/forceps biopsy/aspiration specimens as well as NGS of surgical specimens. In unresectable BTC cases, next-generation sequencing of endoscopic transpapillary specimens can also yield critical genetic information for molecular targeted therapies in addition to CGP with blood specimens. The current BTC treatment strategy consists of (1) the standard chemotherapy with gemcitabine/cisplatin and PD-1/PD-L1/CTLA-4 inhibitors followed by (2) the second-line targeted therapies such as *FGFR2*, *IDH1*, *BRAF*, and *NTRK* based on CGP with NGS (Table 4). Several clinical trials which target alterations of *KRAS* (for all mutations: ClinicalTrials.gov ID: NCT05874414 with combination of GNS561, PPT1 (palmitoyl-protein thioesterase 1) inhibitor and Trametinib, MEK inhibitor; NCT06607185 with the Pan-*KRAS* Inhibitor) and *TP53* (for Y220C mutation) are currently being conducted for solid tumors including CCA [55]. Future validation studies for genomic testing and technical evolution would yield large and evolutionary changes in the early diagnosis and highly targeted therapy of early BTC, and in some cases, biliary intraepithelial lesions.

## Figures and Tables

**Table 1:** Previous reports on gene panel analysis of biliary tract cancer using next-generation sequencing of brushing specimens.

Year	1st Author	BTC Patient No. Control No.	Panel Gene No.	Detected MAF/VAF	DNA Extraction Success, % (No.)	Amount of DNA Obtained
2016	Dudley JC	11 43	39	≥5%	100 (11/11)	ND
2020	Singhi AD	41 ‡ 70	28	≥3%	100 ‡	0.08 to 27.45 ng/μL (mean, 3.17 ng/μL; median, 1.61 ng/μL) ‡
2020	Rosenbaum MW	58 30	39	≥5%	ND	ND
2020	Harbhajanka A	9 51	723	≥2% (≥0.1%)	100	ND
2022	Scheid JF	4 56	Ver. 1, 39 Ver. 2, 116	≥5%	100 (60/60)	ND
2023	Kamp EJCA	20 20	14	≥0.1%	87 (20/23)	Pursued 50 ng
2024	Boyd S	18 8	50	>1–2%	ND	ND
2024	Park W	8 || 11	161 (Bile, 50)	≥3% (Bile, ≥0.3%)	62 (8/13)	ND || (Bile cfDNA ≥ 20 ng, range 1.3 to 20 ng)
**Alteration No./Gene No. †**	**Alteration Incidence** **in Cancer Cases**	**SN**	**SP**	**PPV**	**NPV**	**ACC**
18/10	6/11 (Control: 1/43)	55	98	86	89	89
47/7 ‡	26/41 ‡ (Control: 0/70)	63 ‡	100 ‡	100 § (All)	63 § (All)	86 § (All)
ND	58/58	100	73	88	100	91
15/9	8/9	89	100	100	98	98
25/11	4/4 (PSC control: 6/56)	100	89	40	100	90
22/7	15/20 (PSC control: 4/20)	75	85	79	76	78
27/7	13/18	72	88	ND	ND	ND
21/12 ||	8/8 || (Bile: 7/8)	100 || (Bile 88)	82 ||	80 ||	100 ||	90 ||

ACC, accuracy; BTC, biliary tract cancer; ND, not described; MAF, mutant allele frequency; NPV, negative predictive value; PPV, positive predictive value; PSC, primary sclerosing cholangitis; SN, sensitivity; SP, specificity; VAF, variant allele frequency. † Alteration No. refers to the total number of alterations identified in all cancer patients, while gene No. refers to the number of different genes in which those alterations were identified. ‡ The values are based on data from brushing specimens alone of 41 patients who underwent brushing or both brushing and biopsy. § The values are based on data from both brushing and biopsy specimens of all patients who underwent brushing/biopsy or both brushing and biopsy. || The values are based on data from brushing specimens alone of eight patients who underwent both brushing and biopsy.

**Table 2:** Previous reports on gene panel analysis of biliary tract cancer using next-generation sequencing of forceps biopsy specimens.

Year	1st Author	BTC Patient No. Control No.	Panel Gene No.	Detected MAF/VAF	DNA Extraction Success, % (No.)	Amount of DNA Obtained
2018	Bankov K	16 16	41	17.7% (range, 4.8–79.9)	100 (16/16)	All >10 ng
2020	Singhi AD	90 ‡ 70	28	≥3%	100 ‡	0.53 to 35.71 ng/μL (mean, 6.42 ng/μL; median, 4.22 ng/μL) ‡
2024	Fukuda S	350	124 or 324	ND	ND	ND
2025	Vasuri F	6 6	20	ND	92 12/13	All >10 ng
**Alteration No./Gene No. †**	**Alteration Incidence** **in Cancer Cases**	**SN**	**SP**	**PPV**	**NPV**	**ACC**
51/20	14/16 (Control: /)	88	100	ND	ND	ND
118/18 ‡	64/90 ‡ (Control: 0/70)	71 ‡	100 ‡	100 § (All)	63 § (All)	86 § (All)
ND	ND	ND	ND	ND	ND	ND
11/7	5/6	83	100	100	86	92

ACC, accuracy; BTC, biliary tract cancer; ND, not described; MAF, mutant allele frequency; NPV, negative predictive value; PPV, positive predictive value; SN, sensitivity; SP, specificity; VAF, variant allele frequency. ‡ The values are based on data from brushing specimens alone of 90 patients who underwent brushing or both brushing and biopsy. † Alteration No. refers to the total number of alterations identified in all cancer patients, while gene No. refers to the number of different genes in which those alterations were identified. § The values are based on data from both brushing and biopsy specimens of all patients who underwent brushing/biopsy or both brushing and biopsy.

**Table 3:** Previous reports on gene panel analysis of biliary tract cancer using next-generation sequencing of bile specimens.

Year	1st Author	BTC Patient No. Control No.	Panel Gene No.	Detected MAF/VAF	DNA Extraction Success, % (No.)	Amount of DNA Obtained
2018	Kinugasa H	24 19	49	≥5%	100 (24/24)	ND
2021	Driescher C	4 23	50	≥1%	100	ND
2022	Nagai K	27 8	50	≥2%	100	2.4 to 715 ng/μL (after processing)
2022	Arechederra M	42 13	52	≥0.15%	100	886.10 ± 182.3 ng/mL
2024	Miura Y	43 29	60	ND	ND	Bile 993.3 ng/mL (IQR, 254.4–3360)
2024	Ito S	200	7	>0.005–0.1%	100 (20/20)	79.7 to 6165 ng/μL (after processing)
2025	Bardhi O	20 § 19	28	≥3%	100 (23/23)	ND
2025	Arechederra M	4 59 (PSC)	52	≥0.1%	100 (63/63)	Bile 5.7 μg/mL (range 0.1–70.8 μg/mL) (All cohort)
**Alteration No./Gene No. †**	**Alteration Incidence** **in Cancer Cases**	**SN**	**SP**	**PPV**	**NPV**	**ACC**
17/4	14/24 (Control: 0/19)	58	100	100	66	ND
13/5	4/4 (Control: 0/23)	100	100	100	100	100
26/12	15/27	56	75	88	33	60
116/18	42/42	100	69	91	100	92
83/34	25/43 (PSC control: 6/29)	47	79	77	50	60
20/7	12/20	60	ND	ND	ND	ND
ND	ND	70 §	96 §	88 §	89 §	88 §
5/5	3/4 (PSC control: 20/59)	75 ^‡^	66 ^‡^	13 ^‡^	98 ^‡^	67 ^‡^

ACC, accuracy; BTC, biliary tract cancer; ND, not described; MAF, mutant allele frequency; NPV, negative predictive value; PPV, positive predictive value; PSC, primary sclerosing cholangitis; SN, sensitivity; SP, specificity; VAF, variant allele frequency. † Alteration No. refers to the total number of alterations identified in all cancer patients, while gene No. refers to the number of different genes in which those alterations were identified. ^‡^ The values were calculated using 63 patients with PSC as a control group representing benign cases. § The values are based on data from bile or brushing specimens of 20 patients who underwent brushing or bile aspiration; whether the data were based on bile or brushing specimens was not described.

**Table 4:** Mutated genes and the matched molecular targeted drugs.

Mutated Genes	Drug	Drug Class	Approved/Off-Label
*FGFR2*	Pemigatinib/ Futibatinib	FGFR inhibitors	Approved
*IDH1*	Ivosidenib	Inhibitor of isocitrate dehydrogenase 1	Approved
*BRAF*	Dabrafenib/ Trametinib	BRAF/MEK inhibitor	Approved
*NTRK*	Larotrectinib/ Entrectinib	TRK inhibitors	Approved
*HER2*	Trastuzumab/ Pertuzumab	Anti-HER2 antibody	Off-label
*RET*	Pralsetinib/ Selpercatinib	Selective inhibitor of RET receptor tyrosine kinase	Off-label
*NRG-1*	Zenocutuzumab	Anti-HER2/HER3 antibody	Off-label
*MDM2*	Brigimadlin/ Milademetan	MDM2–p53 antagonist	Off-label
*BRCA1/2*	Olaparib/ Niraparib	Poly adenosine diphosphate-ribose polymerase inhibitors	Off-label

## Data Availability

No new data were created or analyzed in this study. Data sharing is not applicable to this article.

## Supplementary Materials

The following supporting information can be downloaded at https://www.mdpi.com/article/10.3390/diagnostics16101516/s1: Table S1: Previous reports on gene panel analysis of biliary tract cancer using next-generation sequencing of brushing specimens; Table S2: Previous reports on gene panel analysis of biliary tract cancer using next-generation sequencing of forceps biopsy specimens; Table S3: Previous reports on gene panel analysis of biliary tract cancer using next-generation sequencing of bile specimens.
