# Supplementary material for: Comprehensive Gene Panel Analysis of Biliary Tract Cancer Using Next-Generation Sequencing of Endoscopic Transpapillary Brushing/Biopsy/Aspiration Specimens: A Narrative Review
Source: Diagnostics (Basel). 2026 May 16;16(10):1516. doi: 10.3390/diagnostics16101516 (PMC13206024; doi:10.3390/diagnostics16101516)
Supplement: Supplementary file 1 [file diagnostics-16-01516-s001.zip › diagnostics-4275446-supplementary.pdf]

Table S1. Previous reports on gene panel analysis of biliary tract cancer using next-generation sequencing of brushing specimens.

Year	1st Author	BTC Patient No.<br>Control No.	Cancer type (No.)<br>ICC/ECC/GC/AC	Biliary stricture site*<br>Intrah/Perih/Dis/Amp	Panel gene No.	NGS system	Detected MAF/VAF	DNA extraction success, % (No.)
2016	Dudley JC	11<br>43	ICC or ECC or GC 11	ND	39	Illumina MiSeq (Illumina)	≥ 5%	100<br>(11/11)
2020	Singhi AD	41 ‡<br>70	9/24/7/1	9/Perih + Dis 31/0/1	28	Ion Proton™ System (Thermo Fisher Scientific)	≥ 3%	100 ‡
2020	Rosenbaum MW	58<br>30	BTC 58*	ND	39	Illumina MiSeq (Illumina)	≥ 5%	ND
2020	Harbhajanka A	9<br>51	ICC or ECC 8/1/0	ND	723	Ion Torrent PGM (or Ion GeneStudio) (Thermo Fisher Scientific)	≥ 2% (≥ 0.1%)	100
2022	Scheid JF	4<br>56	1/3/0/0	1/2/1/0	Ver. 1, 39<br>Ver. 2, 116	Illumina MiSeq (V1) or NextSeq (V2) instruments (Illumina)	≥ 5%	100<br>(60/60)
2023	Kamp EJCA	20<br>20	0/20/0/0	0/13/7/0	14	Ion S5 System on Ion 540 chips (Thermo Fisher Scientific)	≥ 0.1%	87<br>(20/23)
2024	Boyd S	18<br>8	3/14/1/0	3/ Perih + Dis 15	50	Ion Torrent Proton and S5 Prime System (Thermo Fisher Scientific)	> 1–2%	ND
2024	Park W	8‖<br>11	0/8/0/0	0/4/4/0	161 (Bile, 50)	Ion S5 XL sequencer (Thermo Fisher Scientific)	≥ 3%<br>(Bile, ≥ 0.3%)	62<br>(8/13)

Table S1. continued.

Amount of DNA obtained	Alteration No./ gene No. †	Alteration incidence in cancer cases	SN	SP	PPV	NPV	ACC
ND	18/10	6/11<br>(Control: 1/43)	55	98	86	89	89
0.08 to 27.45 ng/uL (mean, 3.17 ng/uL; median, 1.61 ng/uL) ‡	47/7 ‡	26/41 ‡<br>(Control: 0/70)	63 ‡	100 ‡	100 §<br>(All)	63 §<br>(All)	86 §<br>(All)
ND	ND	58/58	100	73	88	100	91
ND	15/9	8/9	89	100	100	98	98
ND	25/11	4/4<br>(PSC control: 6/56)	100	89	40	100	90
Pursued 50 ng	22/7	15/20<br>(PSC control: 4/20)	75	85	79	76	78
ND	27/7	13/18	72	88	ND	ND	ND
ND|| (Bile cfDNA ≥ 20ng, range 1.3 to 20 ng)	21/12||	8/8|| (Bile: 7/8)	100|| (Bile 88)	82||	80||	100||	90||

AC, ampullary cancer; ACC, accuracy; BTC, biliary tract cancer; ECC, extrahepatic cholangiocarcinoma; GC, gallbladder cancer; ICC, intrahepatic cholangiocarcinoma; Intrah/Perih/Dis/Amp, intrahepatic/perihilar/distal/ampullary; ND, not described; MAF, mutant allele frequency; NGS, next-generation sequencing; NPV, negative predictive value; PPV, positive predictive value; PSC, primary sclerosing cholangitis; SN, sensitivity; SP, specificity; VAF, variant allele frequency.

*Malignant biliary stricture sites alone were assessed. †Alteration No. refers to the total number of alterations identified in all cancer patients, while gene No. refers to the number of different genes in which those alterations were identified. ‡The values are based on data from brushing specimens alone of 41 patients who underwent brushing or both brushing and biopsy. §The values are based on data from both brushing and biopsy specimens of all patients who underwent brushing/biopsy or both brushing and biopsy. ||The values are based on data from brushing specimens alone of 8 patients who underwent both brushing and biopsy.

Table S2. Previous reports on gene panel analysis of biliary tract cancer using next-generation sequencing of forceps biopsy specimens.

Year	1st Author	BTC Patient No.<br>Control No.	Cancer type (No.)<br>ICC/ECC/GC/AC	Biliary stricture site*<br>Intrah/Perih/Distal/Amp	Panel gene No.	NGS system	Detected MAF/VAF	DNA extraction success, % (No.)
2018	Bankov K	16<br>16	4/9/0/3	4/3/6/3	41	Illumina NextSeq500 (Illumina)	17.7%<br>(range, 4.8–79.9)	100<br>(16/16)
2020	Singhi AD	90 ‡<br>70	15/60/6/7	15/Perih + Dis 66/7	28	Ion Proton™ System (Thermo Fisher Scientific)	≥ 3%	100 ‡
2024	Fukuda S	35<br>0	1/24/5/5	1/25/4/5	124 or 324	ND	ND	ND
2025	Vasuri F	6<br>6	0/6/0/0	0/3/10/0	20	ND	ND	92<br>12/13

Table S2. continued.

Amount of DNA obtained	Alteration No./gene No. †	Alteration incidence in cancer cases	SN	SP	PPV	NPV	ACC
All > 10 ng	51/20	14/16<br>(Control: /)	88	100	ND	ND	ND
0.53 to 35.71 ng/uL (mean, 6.42 ng/uL; median, 4.22 ng/uL) ‡	118/18 ‡	64/90 ‡<br>(Control: 0/70)	71 ‡	100 ‡	100 §<br>(All)	63 §<br>(All)	86 §<br>(All)
ND	ND	ND	ND	ND	ND	ND	ND
All > 10 ng	11/7	5/6	83	100	100	86	92

AC, ampullary cancer; ACC, accuracy; BTC, biliary tract cancer; ECC, extrahepatic cholangiocarcinoma; GC, gallbladder cancer; ICC, intrahepatic cholangiocarcinoma; Intrah/Perih/Dis/Amp, intrahepatic/perihilar/distal/ampullary; ND, not described; MAF, mutant allele frequency; NGS, next-generation sequencing; NPV, negative predictive value; PPV, positive predictive value; PSC, primary sclerosing cholangitis; SN, sensitivity; SP, specificity; VAF, variant allele frequency.

*Malignant biliary stricture sites alone were assessed. ‡The values are based on data from brushing specimens alone of 90 patients who underwent brushing or both brushing and biopsy. †Alteration No. refers to the total number of alterations identified in all cancer patients, while gene No. refers to the number of different genes in which those alterations were identified.

§The values are based on data from both brushing and biopsy specimens of all patients who underwent brushing/biopsy or both brushing and biopsy.

Table S3. Previous reports on gene panel analysis of biliary tract cancer using next-generation sequencing of bile specimens.

Year	1st Author	BTC Patient No.<br>Control No.	Cancer type (No.)<br>ICC/ECC/GC/AC	Biliary stricture site*<br>Intrah/Perih/Distal/Amp	Panel gene No.	NGS system	Detected MAF/VAF	DNA extraction success, % (No.)
2018	Kinugasa H	24<br>19	0/0/24/0	0/Perih + Dis 24/0	49	Illumina MiSeq (Illumina)	≥ 5%	100<br>(24/24)
2021	Driescher C	4<br>23	ICC or ECC 4/0/0	Intrah + Perih + Dis 4	50	Ion S5™ System (Thermo Fisher Scientific)	≥ 1%	100
2022	Nagai K	27<br>8	2/24/1/0	2/17/8/0	50	Ion Personal Genome Machine System (Life Technologies)	≥ 2%	100
2022	Arechederra M	42<br>13	3/38/1/0	3/16/I23/0	52	Ion S5™ System (Thermo Fisher Scientific)	≥ 0.15%	100
2024	Miura Y	43<br>29	4/29/10/0	4/24/15/0	60	Ion Proton™ System (Thermo Fisher Scientific)	ND	ND
2024	Ito S	20<br>0	1/13/3/3	1/9/7/3	7	QX200 and AutoDG ddPCR Systems (Bio-Rad)	> 0.005–0.1%	100<br>(20/20)
2025	Bardhi O	20§<br>19	ICC or ECC 20/0/0	Intrah + Perih 2/Perih + Dis 18	28	Ion Proton™ System (Thermo Fisher Scientific)	≥ 3%	100<br>(23/23)
2025	Arechederra M	4<br>59 (PSC)	1/3/0/0	1/3/0/0	52	Ion S5™ System (Thermo Fisher Scientific)	≥ 0.1%	100<br>(63/63)

Table S3. continued.

Amount of DNA obtained	Alteration No./gene No. †	Alteration incidence in cancer cases	SN	SP	PPV	NPV	ACC
ND	17/4	14/24 (Control: 0/19)	58	100	100	66	ND
ND	13/5	4/4 (Control: 0/23)	100	100	100	100	100
2.4 to 715 ng/μL (after processing)	26/12	15/27	56	75	88	33	60
886.10 ± 182.3 ng/mL	116/18	42/42	100	69	91	100	92
Bile 993.3 ng/mL (IQR, 254.4–3360)	83/34	25/43 (PSC control: 6/29)	47	79	77	50	60
79.7 to 6165 ng/μL (after processing)	20/7	12/20	60	ND	ND	ND	ND
ND	ND	ND	70 §	96 §	88 §	89 §	88 §
Bile 5.7 μg/mL (range 0.1–70.8 μg/mL) (All cohort)	5/5	3/4 (PSC control: 20/59)	75‡	66‡	13‡	98‡	67‡

AC, ampullary cancer; ACC, accuracy; BTC, biliary tract cancer; ECC, extrahepatic cholangiocarcinoma; GC, gallbladder cancer; ICC, intrahepatic cholangiocarcinoma; Intrah/Perih/Dis/Amp, intrahepatic/perihilar/distal/ampullary; ND, not described; MAF, mutant allele frequency; NGS, next-generation sequencing; NPV, negative predictive value; PPV, positive predictive value; PSC, primary sclerosing cholangitis; SN, sensitivity; SP, specificity; VAF, variant allele frequency

*Malignant biliary stricture sites alone were assessed.†Alteration No. refers to the total number of alterations identified in all cancer patients, while gene No. refers to the number of different genes in which those alterations were identified. ‡The values were calculated using 63 patients with PSC as a control group representing benign cases. §The values are based on data from bile or brushing specimens of 20 patients who underwent brushing or bile aspiration; whether the data were based on bile or brushing specimen was not described.
